# Supplementary material for: Application of the flipped classroom model based on Bloom’s Taxonomy of Educational Objectives in endodontics education for undergraduate dental students
Source: PeerJ. 2025 Jan 29;13:e18843. doi: 10.7717/peerj.18843 (PMC11786706; doi:10.7717/peerj.18843)
Supplement: Supplemental Information 2 [file peerj-13-18843-s002.docx]

Questionnaire on the effectiveness of teaching with the flipped classroom method in the bachelor's degree program in dentistry

Department of Endodontics, Guanghua School of Stomatology, Sun Yat-sen University, China

Dear Classmates：

First of all, thank you for completing this questionnaire. The results are crucial for us to improve our teaching methods. On behalf of the faculty, we would like to thank you for your cooperation. In the field of Endodontics, specifically in root canal therapy, which is characterized by a multitude of complex concepts and challenges in comprehension, we are actively seeking and exploring innovative teaching methods to simplify the course material, enhance interactively, and make the learning experience more enjoyable. We have created this questionnaire to investigate the effectiveness of teaching. Please read the questionnaire carefully and try to select answers that are realistic.

Name： Genders： Grade： Classes：

1. At the end of this chapter, you have evaluated the content of this chapter on root canal therapy as follows (10 out of 10, 6 passed).

10 9 8 7 6 5 4 3 2 1

2. At the end of this chapter, your knowledge of the topic "overview of the development of root canal therapy and case selection" will be assessed as follows (10 out of 10, 6 passed).

10 9 8 7 6 5 4 3 2 1

3. At the end of this chapter, your knowledge of the anatomy of the medullary cavity is assessed as follows (10 out of 10, 6 passed).

10 9 8 7 6 5 4 3 2 1

4. At the end of this chapter, your knowledge of "root canal preparation and sterilization" will be assessed as follows (10 out of 10, 6 passed).

10 9 8 7 6 5 4 3 2 1

5. How would you rate your knowledge of "root canal filling" in this chapter? (10 out of 10, 6 passed).

10 9 8 7 6 5 4 3 2 1

6. At the end of this chapter, your knowledge of "strategies for prevention and treatment of complications in root canal therapy" will be assessed as follows (10 out of 10, 6 passed).

10 9 8 7 6 5 4 3 2 1

7. The syllabus will help you to understand the aims and key points of the chapter.

a) □Very agree b) □Agree c) □Unsure d) □Disagree e) □Very disagree

8. The syllabus will help you understand the ethical, and humanistic teaching objectives of the chapter.

a) □Very agree b) □Agree c) □Unsure d) □Disagree e) □Very disagree

9. Learning and mastering the contents of this chapter will facilitate your growth as a doctor with medical ethics, competence and cordiality.

a) □Very agree b) □Agree c) □Unsure d) □Disagree e) □Very disagree

10. This course will awaken your interest in Endodontics.

a) □Very agree b) □Agree c) □Unsure d) □Disagree e) □Very disagree

11. Are you satisfied with the teaching mode in this chapter?

a) □Very agree b) □Agree c) □Unsure d) □Disagree e) □Very disagree

12. Were you satisfied with the way you reviewed this chapter before class?

a) □Very agree b) □Agree c) □Unsure d) □Disagree e) □Very disagree

13. Are you satisfied with this part of the curriculum?

a) □Very agree b) □Agree c) □Unsure d) □Disagree e) □Very disagree

14. Are you satisfied with the duration of the course (2 credit hours)

a) □Very agree b) □Agree c) □Unsure d) □Disagree e) □Very disagree

15. The total time you spent studying before class was minutes.

16. The total time you spent reviewing after school was minutes.

17. If you have any further comments or suggestions on the teaching chapter, please fill in this form
