# Supplementary material for: Application of the flipped classroom model based on Bloom’s Taxonomy of Educational Objectives in endodontics education for undergraduate dental students
Source: PeerJ. 2025 Jan 29;13:e18843. doi: 10.7717/peerj.18843 (PMC11786706; doi:10.7717/peerj.18843)
Supplement: Supplemental Information 3 [file peerj-13-18843-s003.docx]

口腔医学专业本科牙髓病学教学效果的调查问卷

中山大学光华口腔医学院牙体牙髓病学教研室

亲爱的同学：

首先感谢您回答这份问卷，结果对我们改革教学方法有很重要的作用。我们代表教研室对您的合作表示感谢。在牙体牙髓病学《根管治疗术》这一章节里存在知识点多与理解困难的特点，我们正在寻求并研究教学的新模式，使得这门课程更容易理解和增加学习的互动和乐趣。我们建立此问卷对《根管治疗术》的教学效果进行调查。请认真阅读问卷，并尽量选出符合实际情况的答案。

姓名： 性别： 年级： 班级：

1.通过本章的学习，您对本章节《根管治疗术》的内容掌握程度评分为（10分为满分，6分为及格）

10 9 8 7 6 5 4 3 2 1

2. 通过本章节的学习，您对“根管治疗术的发展概况及病例选择”的相关知识掌握程度的评分为（10分为满分，6分为及格）

10 9 8 7 6 5 4 3 2 1

3. 通过本章节的学习，您对“髓腔的解剖形态”的相关知识掌握程度的评分为（10分为满分，6分为及格）

10 9 8 7 6 5 4 3 2 1

4. 通过本章节的学习，您对“根管预备与消毒”的相关知识掌握程度的评分为（10分为满分，6分为及格）

10 9 8 7 6 5 4 3 2 1

5. 通过本章节的学习，您对“根管充填”的相关知识掌握程度的评分为（10分为满分，6分为及格）

10 9 8 7 6 5 4 3 2 1

6. 通过本章节的学习，您对“根管治疗并发症的预防与处理策略”的相关知识掌握程度的评分为（10分为满分，6分为及格）

10 9 8 7 6 5 4 3 2 1

7. 教学大纲可以帮助您了解本章节的教学目标与要点

a)□十分同意 b)□同意 c)□不确定 d)□不同意 e)□非常不同意

8. 教学大纲可以帮助您了解本章节的医德医风等与人文教学目标

a)□十分同意 b)□同意 c)□不确定 d)□不同意 e)□非常不同意

9. 本章节内容的学习与掌握有利于您成长为一名有医德、有医术、有温度的医生

a)□十分同意 b)□同意 c)□不确定 d)□不同意 e)□非常不同意

10. 本课程能激发您对牙髓病学的学习兴趣

a)□十分同意 b)□同意 c)□不确定 d)□不同意 e)□非常不同意

11. 您对本章节课堂教学模式是否满意

a)□十分同意 b)□同意 c)□不确定 d)□不同意 e)□非常不同意

12. 您对本章节课前预习方式是否满意

a)□十分同意 b)□同意 c)□不确定 d)□不同意 e)□非常不同意

13. 您对本章节教学大纲是否满意

a)□十分同意 b)□同意 c)□不确定 d)□不同意 e)□非常不同意

14. 您对课堂时间长度（2学时）是否满意

a)□十分同意 b)□同意 c)□不确定 d)□不同意 e)□非常不同意

15. 您课前预习时间长度累计为 分钟

16. 您课后复习时间长度累计为 分钟

17. 如果对章节教学有其他意见或者建议，请填写 。
